# BRCA Mutations Increase Fertility in Families at Hereditary Breast/Ovarian Cancer Risk

**DOI:** 10.1371/journal.pone.0127363

**Published:** 2015-06-05

**Authors:** Fabrice Kwiatkowski, Marie Arbre, Yannick Bidet, Claire Laquet, Nancy Uhrhammer, Yves-Jean Bignon

**Affiliations:** 1 Centre Jean Perrin, Laboratoire d'Oncologie Moléculaire, 63011, Clermont-Ferrand, France; 2 Université Blaise Pascal—Laboratoire de Mathématiques, UMR 6620—CNRS, Campus des Cézeaux—BP, 80026–63171, Aubière cedex, France; 3 Université Clermont Auvergne, Université d'Auvergne, BP 10448, F-63000, Clermont-Ferrand, France; University of Hawaii Cancer Center, UNITED STATES

## Abstract

**Background:**

Deleterious mutations in the BRCA genes are responsible for a small, but significant, proportion of breast and ovarian cancers (5 - 10 %). Proof of *de novo* mutations in hereditary breast/ovarian cancer (HBOC) families is rare, in contrast to founder mutations, thousands of years old, that may be carried by as much as 1 % of a population. Thus, if mutations favoring cancer survive selection pressure through time, they must provide advantages that compensate for the loss of life expectancy.

**Method:**

This hypothesis was tested within 2,150 HBOC families encompassing 96,325 individuals. Parameters included counts of breast/ovarian cancer, age at diagnosis, male breast cancer and other cancer locations. As expected, well-known clinical parameters discriminated between BRCA-mutated families and others: young age at breast cancer, ovarian cancer, pancreatic cancer and male breast cancer. The major fertility differences concerned men in BRCA-mutated families: they had lower first and mean age at paternity, and fewer remained childless. For women in BRCA families, the miscarriage rate was lower. In a logistic regression including clinical factors, the different miscarriage rate and men's mean age at paternity remained significant.

**Results:**

Fertility advantages were confirmed in a subgroup of 746 BRCA mutation carriers and 483 non-carriers from BRCA mutated families. In particular, female carriers were less often nulliparous (9.1 % of carriers versus 16.0 %, p = 0.003) and had more children (1.8 ± 1.4 SD versus 1.5 ± 1.3, p = 0.002) as well as male carriers (1.7 ± 1.3 versus 1.4 ± 1.3, p = 0.024).

**Conclusion:**

Although BRCA mutations shorten the reproductive period due to cancer mortality, they compensate by improving fertility both in male and female carriers.

## Introduction

Dominant deleterious mutations in a population should be suppressed unless they have a compensating effect on fertility or are expressed only after the fertile period. Highly penetrant deleterious mutations are subject to selection pressure [1]; those affecting young people without conferring a fertility advantage will therefore often be *de novo* mutations. Mutations in the tumor suppressor genes TP53 or pRb, where cancer often develops in childhood, follow this pattern [2]. Deleterious mutations in the BRCA genes, responsible for hereditary breast/ovarian cancers (HBOC), only partially follow this model. The majority are unique to a family (cf http://research.nhgri.nih.gov/bic/ and http://www.umd.be/BRCA1/, for example), though it is usually not possible to determine in what person it first occurred, and reports of *de novo* mutations are quite rare [3–4]. But this latter point should nowadays be perspectived: frenquency of germline *de novo* mutations has been found higher in recent studies where a rate of 3.5 to 4 mutations per individual is estimated (mostly from male genitors) [5], and with an increasing mutational risk with age [6]. This is in accordance with the French UMD-BRCA1/BRCA2 database where about half of the mutations were unique to a family (thus possibly recent)—respectively 53% and 63% of deleterious BRCA1 and BRCA2 mutations—while 18 other mutations occurred in at least 8 families and two in more than 130 [7]. The haplotypes of many recurring mutations have a common ancestral origin; although a few have occurred more than once, some of those segregating in specific populations are known to be thousands of years old [8–10]. BRCA mutations thus appear to be a mix of rare private mutations, some of which may be recent, and more common mutations passed down through numerous generations.

The age at which BRCA mutation carriers develop cancer overlaps the reproductive period, so there should be some mechanism by which mutations persist in the population. Several studies have noted that BRCA mutation carriers have higher parity than non-carriers, suggesting a positive effect on female fertility [11]. Others, in contrast, have associated BRCA mutation with reduced ovarian function [12], or with voluntarily reduced reproduction [13], and still others have found no effect [14]. We thus decided to evaluate fertility outcomes (resulting from reproduction factors possibly unknown) concurrently with known predictive factors for BRCA mutational status, in a large database of 2,936 BRCA and non-BRCA HBOC families. A two-step analysis was realized: first with families considered as a entity, then by individuals grouped according to their known BRCA mutational status.

## Materials and Methods

### Pedigrees

Families were accrued at the oncogenetic consultation of the Centre Jean Perrin in central France from 1988 to 2013, and included a huge majority of Caucasians (≈98%). Information collected for pedigrees comprised extensive notation of the proband's relatives without limitation of the number of generations included as long as cause of death was known: median generation count was 4 and interquartile interval [2; 5]). Also included were cancer location and age at diagnosis, mutations known or discovered subsequently, date of birth, gender, marital status, descendancy, miscarriages, dates of marriage, separation and death.

The database was declared to the French National Informatics & Liberty Committee (CNIL) on May 18^th^ 2011 by the CIL (the local CNIL correspondant) and in accordance with the article R. 1131–2 of the French public health code, counselees signed a special consent enabling the use of their data for reasearch purpose. It was managed using SEM software [15], which also performed statistics and special calculations (age at first/last birth, age at first cancer, rates per family of miscarriage or of childlessness…).

**Fig 1:**
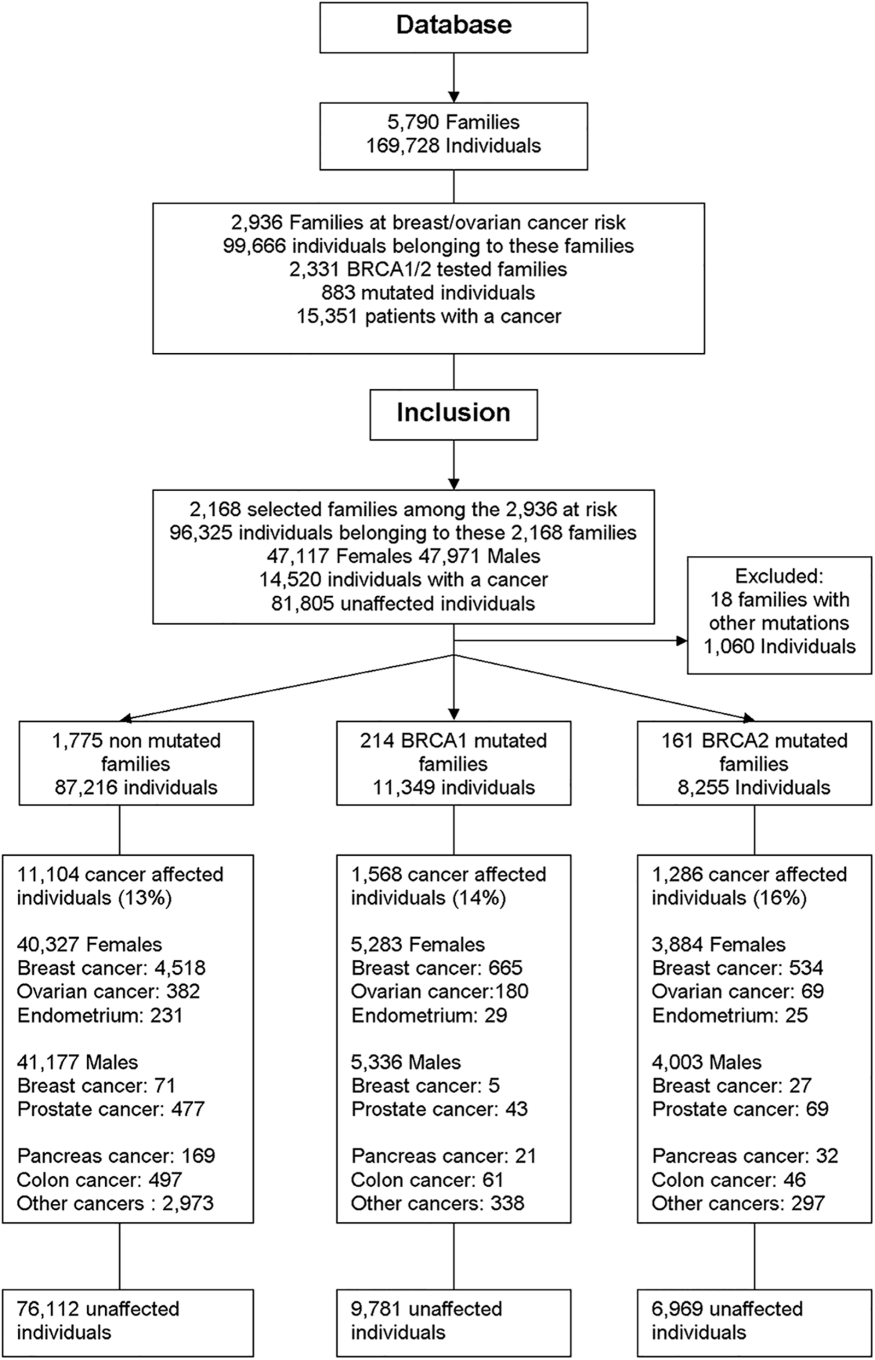
Accrual flowchart of the families and the individuals selected from the Database.

When pedigrees with a breast/ovarian cancer risk were extracted from the database, 2,168 families including 96,325 at-risk individuals were selected. Families needed to contain at least 5 female affiliated members (i.e. ≈ 10 members), otherwise we considered that not enough pedigree information was available to be reliable. BRCA1 mutations were found in 10% of families (214 families; 11,349 members), and BRCA2 mutations in 7% (161 families; 8,255 members). In 1,775 families (87,216 members) no mutation was found: this group is identified hereafter as "no mutation". Eighteen families diagnosed with other mutated genes were excluded (Fig 1): 2,150 families were thus statistically analysed.

The average number of family members was 51.2 ± 35.1 (SD), with on average 23.7 ± 15.7 females, and 24.2 ± 16.6 males per pedigree. Cancer in these families included 5,821 breast cancers (5,718 female and 103 male), 631 ovarian cancers, 285 endometrium, 604 colon, 222 pancreatic, 589 prostatic and 3,608 other location.

### Prediction parameters

Excepted for fertility, relevant parameters were retrieved from published scoring strategies [16–21]. The following cancer locations were included: breast, ovary, endometrium, pancreas, colon and prostate; all other locations were grouped together. Bilateral breast cancers were counted twice. Female breast cancers were categorized by age at diagnosis in 10-year classes: <30, 30–39, 40–49, 50–59, 60–69, ≥70. Triple-negative breast cancers were not considered because their reporting was too recent and incomplete in our database.

Statistical analysis included the following counts:

- Number of breast cancers in families by class of age
- Number of male breast cancers
- Number of cancers detailed by: ovary, endometrium, colon, pancreas, prostate
- Number of cancers of any other type
- Number of persons with multiple cancers
- Number of members per pedigree and number of males and females
- Number of miscarriages
- Average number of children per potential mother (i.e. women with at least a spouse and/or a child and/or age ≥ 40)
- Average mother’s age at first child (if any).

When age at cancer diagnosis was unknown, age at last followup (or of death) was used instead, considering that in older generations, the average time between these events was short. When ages at cancer diagnosis and death were both unknown, we replaced missing values by the average age of family members belonging to the same generation. Age at first child was computed for each woman who had a child, and the average computed by family. Finally, an average number of children was calculated per family, taking into account women with at least one child and/or being in a couple at least once, or being older than 40 years (an age *a priori* sufficient to give birth at least once). We used this strategy because the proportion of young single women could differ between groups and counting them as “zero-child mothers” could bias the results. Inherited risk calculations and other statistics were not applied to spouses: only members exposed to the familial cancer-risk were used in calculations.

### Statistics

Only the branch(es) with cancer risk was entered into the database for > 90% of pedigrees, so calculations were done by entire pedigree. Univariate comparisons with mutation status were performed using Z-test or H-test depending on homoscedasticity and/or normality of distributions. Tests were two-sided and a p value ≤ 0.05 was considered significant. Multivariate analysis to order covariates consisted of backward logistic regressions. The adequacy of models to the data was evaluated with the Hosmer-Lemeshow test. To test the efficacy of scores to predict mutation status, a ROC analysis was performed, and the area under curve (AUC) compared together.

Significant clinical factors predictive for BRCA-mutated status were selected first using univariate analysis, then classified by logistic regression. Fertility parameters associated to the mutation status by univariate analysis to a p-value ≤ 0.10 were then introduced in the logistic regression model concurrently with significant clinical factors.

Comparisons were performed within following groups:

- BRCA mutation versus no-mutation
- BRCA1 mutation versus no-mutation
- BRCA2 mutation versus no-mutation
- BRCA1 mutation versus BRCA2 mutation

## Results

### a) role of standard clinical parameters on BRCA mutation risk

Repartition of main cancer locations according to mutation status of families are exhibited in Table 1. Each group corresponds to members of families where a BRCA mutation was found, without testing the mutation status of each individual.

**Table 1:** Number of cancer locations according to diagnosed mutation (% of members^(*)^).

Cancer location	BRCA1	BRCA2	No mutation	p-value
**Women: Breast**	502 (12.2%)	419 (13.7%)	3,478 (10.7%)	0.00013
**Ovarian**	138 (3.3%)	61 (2.0%)	285 (0.9%)	< 10–7
**Endometrium**	22 (0.5%)	20 (0.7%)	200 (0.6%)	0.91
**Men: Breast**	2 (0.04%)	14 (0.44%)	53 (0.16%)	0.00053
**prostate**	36 (0.8%)	57 (1.8%)	386 (1.1%)	0.026
**Any sex: Colon**	56 (0.7%)	36 (0.6%)	390 (0.9%)	0.59
**Pancreas**	17 (0.2%)	22 (0.4%)	125 (0.3%)	0.02
**Other location**	295 (3.6%)	281(4.5%)	2,684 (6.4%)	0.42
**Multiple location**	76 (1.8%)	70 (2.2%)	570 (1.8%)	0.13
**Any cancer**	1068 (12.9%)	910 (14.6%)	7,601 (11.7%)	0.0001

P-values are associated to 3-group comparisons.

(*) percentages are calculated on numbers of included female or male individuals concerned by the location (for example, only female for ovarian cancers)

**Fig 2:**
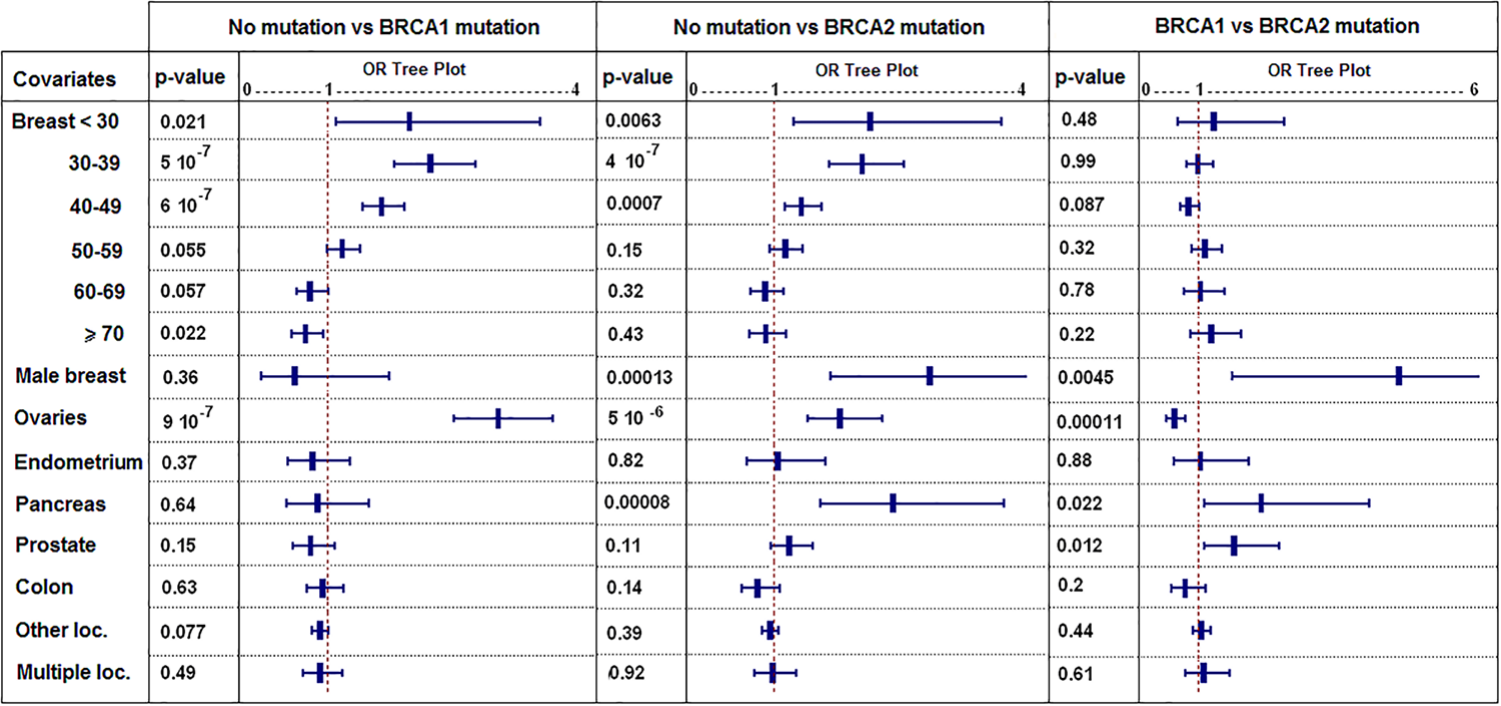
Cancer locations predicting the BRCA mutational risk. (logistic regression: p-values complete the information given by each Odds-Ratio; error bars represent 95%-CI of Odds-Ratios; covariates are cancer locations and "Breast < 30" means female breast cancers occurring before 30 years…).

Logistic regression enabled us to estimate the respective weight of each parameter. Standard clinical factors (Fig 2) predicted both BRCA mutation status (BRCA1 and BRCA2), notably the number of breast cancers occurring before 50 years and ovarian cancers. Pancreatic and male breast cancers were significant for BRCA2. The distinction between a BRCA1 and a BRCA2 mutation depended on four factors: ovarian cancers favored BRCA1 mutations (p = 0.00011) while male breast cancers (p = 0.0045), prostatic cancers (p = 0.012) and pancreatic cancer (p = 0.022) were more frequent in BRCA2 mutated families.

In a global comparison by logistic regression of BRCA1 plus BRCA2 mutated families to no-mutation families, the best fitted model performed slightly better (ROC AUC = 0.73 [0.70–0.76]) than the Eisinger and Manchester predictive scores (AUC = 0.70 [0.33–0.73] for both), but this difference was not significant. Also, Eisinger's score incorporating very few elements (ovarian cancers, female breast cancers depending on 6 classes of age and male breast cancers) performed as well as the Manchester score including 15 parameters (16 if triple-negative breast cancer status is included).

### b) Average fertility characteristics per family

The initial analysis compared whole families where at least one individual was diagnosed with a BRCA mutation to other families. A fertility characteristic for a family corresponds to the average of the characteristic calculated across all its affiliated members.

The miscarriage rate in mutated versus non-mutated families was reduced by 35% (p = 0.015), while other fertility parameters were similar (Table 2). In men from mutated versus non-mutated families, the average number of offspring was slightly lower (p = 0.041), but the reproductive period was advanced by 7 months, with lower overall mean reproduction age (p = 0.0013), earlier first child (p = 0.0023) and earlier last child (p = 0.018).

**Table 2:** Fertility parameters depending on the presence of a BRCA mutation in the family: p-values correspond to tests performed between BRCA1+2 mutated families versus not mutated ones (NM).

Gender	Natality parameter	BRCA1	BRCA2	No mutation (NM)	p-value (BRCA1+2 vs NM)
**Affiliated Women**	**N =**	3,951	2,978	30,134	
	**Number of children**	2.6 ± 0.8	2.6 ± 0.8	2.6 ± 0.8	0.61
	**Sex ratio of children (M/F)**	0.89 ± 0.47	0.89 ± 0.46	0.89 ± 0.53	0.92
	**Age at first child**	25.1 ± 2.3	24.9 ± 2.4	25.2 ± 2.8	0.38
	**Average age at all children**	27.5 ± 2.3	27.6 ± 2.4	27.6 ± 2.7	0.55
	**Age at last child**	30.1 ± 2.8	30.5 ± 3.3	30.3 ± 3.4	0.83
	**Miscarriage**	0.16 ± 0.62	0.16 ± 0.56	0.25 ± 1.02	**0.015**
	**Nulliparous**	1,298 (32.9%)	952	10,819 (35.9%)	0.51
			(32.0%)		
**Affiliated Men**	**N =**	3,381	2,682	23,357	
	**Number of children**	2.6 ± 0.9	2.6 ± 1.0	2.7 ± 1.1	**0.041**
	**Sex ratio of children (M/F)**	0.82 ± 0.51	0.84 ± 0.49	0.82 ± 0.48	0.92
	**Age at first child**	27.5 ± 3.1	27.5 ± 3.2	28.1 ± 3.7	**0.0023**
	**Average age at all children**	30.1 ± 2.9	30.1 ± 3.2	30.7 ± 3.7	**0.0013**
	**Age at last child**	32.7 ± 4.1	32.9 ± 4.1	33.6 ± 4.8	**0.018**
	**No child**	1 246 (36.9%)	971	10,133 (43.4%)	0.73
			(36.2%)		

### c) Per-family multivariate analysis of natality parameters compared to standard clinical factors predictive for BRCA mutations

We first analysed all natality parameters together in order to extract most important ones. These selected parameters were then added to previous models in order to test if they were independently significant. Two natality factors remained significant when we compared BRCA mutated to non-mutated families: the mean age at fatherhood was lower (p = 0.0028), as was the rate of miscarriages in women (p = 0.021) (Table 3).

**Table 3:** Influence of new natality parameters on the risk for BRCA mutation when analyzed concurrently with cancer locations and age at diagnosis.

	BRCA1 or BRCA2 versus no-mutation	BRCA1 alone versus no-mutation	BRCA2 alone versus no-mutation	BRCA1 versus BRCA2
**Men mean age at any birth**	0.94 [0.91; 0.98]	0.94 [0.89; 0.99]	0.94 [0.90; 1.00]	1.00 [0.92; 1.08]
	p = 0.0028	p = 0.013	p = 0.038	p = 0.93
**Miscarriages**	0.80 [0.66; 0.97]	0.81 [0.63; 1.03]	0.81 [0.61; 1.06]	1.12 [0.77; 1.63]
	p = 0.021	p = 0.088	p = 0.12	p = 0.55

First line = Odds-Ratios with 95%-CI; second line = p-value. Usual significant parameters are not reported as they are like in Fig 2.

The odds ratios given in Table 3 signify that one supplementary year of paternal age diminishes by 6% the chances of belonging to a family with a BRCA mutation, and a 1% increase in the miscarriage rate decreases this probability by 0.2%. Odds ratios for both fertility parameters were significant in the global logistic regression analysis (BRCA1 or BRCA2 vs NM column). However, addition of both parameters to the regression model did not improve the overall predictability, as the area under curve of the associated ROC curve remained almost stable at 0.74 [0.71; 0.77]. Neither parameter differentiated BRCA1 risk from BRCA2 risk.

### d) Variation of individual natality characteristics according to known BRCA mutational status

Most members of our pedigrees have not been tested for mutations, notably in oldest generations, although a significant proportion of them must carry the familial mutation. We thus analyzed members with known BRCA mutation status.

Three groups were constituted:

- 583 members tested positive for a BRCA mutation
- 634 members tested negative for a BRCA mutation but belonging to families where a BRCA mutation was found
- 306 members tested negative for a BRCA mutation and belonging to HBOC families where no BRCA mutation was found

The second group is the “ideal” control group (they are very unlikely to carry another mutation favoring cancer) while people belonging to the third group may carry an unknown deleterious mutation (because they were selected for BRCA analysis) that could impact reproductive outcomes. Main differences concerned the average number of children either for men and women (Table 4).

**Table 4:** Fertility parameters in 1,546 BRCA-tested individuals according to their BRCA mutational status and if they belong or not to a BRCA mutated family.

Gender	Natality parameter	BRCA mutated	p-value	Not BRCA mutated but of a mutated family	p-value	No known deleterious mutation diagnosed in the family
		(1)	(1) vs (2)	(2)	(2) vs (3)	(3)
**Women**	N tested	583		364		306
	**Childless**	9.1%	**0.003**	16.0%	0.91	15.7%
	(Nb cases / N’)	(46 / 507)		(47 / 293)		(45 / 287)
	Number of children(*)	1.8 ± 1.4	**0.002**	1.5 ± 1.3	**0.0017**	1.8 ± 1.3
	**Age at first birth**	24.9 ± 4.3	0.97	24.7 ± 4.1	0.94	24.8 ± 4.7
	**Mean age at any birth**	26.9 ± 4.1	0.32	26.6 ± 4.0	0.57	26.8 ± 4.5
	**Age at last birth**	29.2 ± 5.2	0.14	28.6 ± 4.9	0.35	29.0 ± 5.3
	Last—first birth (y) (**)	5.9 ± 4.2	0.24	5.5 ± 3.7	0.96	5.6 ± 4.1
	**Miscarriages reported**	3.1%	0.10	1.4%	0.10	3.4%
**Men**	N tested	163		119		11
	**Childless**	11.3%	0.42	14.9%	0.68	9.1%
	(Nb cases / N’)	(16 / 141)		(14 / 94)		(1 / 11)
	Number of children(*)	1.7 ± 1.3	**0.024**	1.4 ± 1.3	**0.036**	2.2 ± 1.1
	**Age at first birth**	26.6 ± 4.0	0.21	27.3 ± 4.2	0.62	26.6 ± 2.4
	**Mean age at any birth**	28.8 ± 4.1	0.58	29.1 ± 4.0	0.86	29.3 ± 3.5
	**Age at last birth**	31.3 ± 5.4	0.80	31.1 ± 4.9	0.50	32.2 ± 6.0
	Last—first birth (y) (**)	6.1 ± 4.3	0.22	5.6 ± 4.6	0.59	7.0 ± 5.3
**Both gender**	**Childless**	9.6%	**0.0029**	18.8%	0.68	15.4%
	(Nb cases / N’)	(62 / 648)		(61 / 387)		(46 / 298)
	Number of children(*)	1.7 ± 1.4	**0.00013**	1.5 ± 1.3	**0.00012**	1.8 ± 1.3
	**Age at first birth**	25.2 ± 4.5	0.49	25.4 ± 4.3	0.20	24.9 ± 4.7
	**Mean age at any birth**	27.3 ± 4.3	0.70	27.3 ± 4.4	0.71	26.9 ± 4.5
	**Age at last birth**	29.6 ± 5.3	0.22	29.2 ± 5.0	0.99	29.2 ± 5.4
	Last—first birth (y) (**)	5.9 ± 4.3	0.24	5.5 ± 4.0	0.80	5.7 ± 4.1

p-values correspond to comparisons between columns.

(*) including nulliparous members.

(**) for individuals with at least 2 children with known dates of birth.

N’ = number of married/common-law individuals or singles ≥ 40 years old.

(y) years.

Nulliparity (childlessness) corresponded to individuals without child and aged ≥ 40 years, else without child but married/common-law. This age was chosen so that the nulliparity due to young age would not bias outcomes. The rate of childless individuals was reported to the overall number of persons aged over 40 and/or married or common-law with or without children. Childless women were notably rarer among BRCA carriers both when compared to women of second (p = 0.003) and of third group (p = 0.005)

The reproductive period was slightly longer for women and men carrying a BRCA mutation (respectively 5.9 versus 5.5 years and 6.1 versus 5.6) but without significance. Offspring in females was higher (p = 0.002) and was likely related to the excess of nulliparous women (6.7%, p = 0.019) among non-carrier family members. Male carriers also had on average more children than non-carrier family members (p = 0.024).

Comparison between first and last groups of Table 4 were not detailed: they evidenced no significant difference excepted for the rate of childless women noted above.

Comparisons between groups two and three exhibited almost as many differences as between the first and the second groups. The exception noted above, concerned the rate of childless women that was similar for all non BRCA mutated individuals and close to 16%.

## Discussion

BRCA mutations seem to provide fertility advantages that compensate for increased cancer risk and mortality, mainly through an increased number of children, possibly related to a lower rate of childlessness and a longer interval between first and last child. Unexpectedly, fertility differences, calculated on families, were more significant among males than among females. Aside from the childlessness rate, these outcomes confirm those reported by Smith *et al*. in a case-control study of 181 BRCA mutation carriers from 49 kindreds versus 1830 controls [11], all having at least one child, and born before 1930 to avoid the influence of modern birth control. We tested their hypothesis, comparing only persons born before or after 1930, but no major divergence was found between older and more recent cohorts in our population (data not shown). Our results and Smith’s partly contradict the conclusions of a study of 96 female mutation carriers, 164 non-carrier cases and 331 controls, which did not show any fertility increase related to BRCA mutations, but which also did not study male fertility [13]. This last study observed a lower male/female ratio for the offspring of female BRCA mutation carriers, which could not be confirmed in our population (data not shown). In a large North American study [14], no fertility differences were found between 2,254 female BRCA carriers and 764 controls from mutated families. But their population was rather recent and young, and the use of contraceptive generalized. The absence of studies concerning male carriers may have hidden their role in the maintenance of deleterious BRCA alleles in the population.

Although observations for tested individuals may appear more reliable than those for whole families, this could be subject to bias. Tested individuals are usually younger than other adults of their families (notably, members of preceding generations are no longer available for direct study). Later generations are more subject to recent birth control measures, as well as social changes in desired family size and delay before having a first child, minimizing small differences in overall reproductive capacity. That is why we also reported statistics based on families including all available generations. The disadvantage of this latter approach is that not all members of BRCA-mutated families are carriers. A similar phenomenon happens in families where no BRCA mutation has been diagnosed. Mutations in other genes are likely to be present in many of these families, and as shown in Table 4, members from the no-familial-mutation group (3) are often closer to BRCA carriers (1) than to non-carrier family members (2): this is in particular true for the average number of children.

Fertility advantages may come from various causes. Some may be strictly biologic, for example a mutation that could play a role on sex hormones production, on an earlier onset of fertility, on the sperm quality, or that could limit the in-utero rejection of a malformed embryo. Apart from the evident influences of the cultural context, it is also possible that some mutated genes could impact behavioral aspects that modify *in fine* the reproductive outcomes. This is why we studied various dimensions of the fertility, in particular the onset and the duration of the reproduction period, the miscarriage rate, and the “celibacy” rate (equivalent to the childlessness rate in our study as 163 of the 169 childless BRCA tested individuals were single). We detail this different factors.

The fertility advantages we observed in mutation carriers occur earlier in life than the age at which breast or ovarian cancer usually develops, and they play a protective role against cancer, since a high number of children and an earlier first child for females are known to reduce breast cancer risk, partly in relation to breast-feeding duration [22–24]. This may explain why differences are stronger in males than in females as these adjustments do not exist for the former. This point is also important for males as later births are more exposed to *de novo* germline mutations, thus probably to congenital malformations [3].

The lower proportion of childless carriers was also an interesting result, and does not seem attributable to a difference in the way pedigrees were collected because we studied only families including at least five at-risk members. If confirmed, this may indicate that genetic profiles, besides cultural environment, could influence reproductive behavior. One might suggest that individual reproductive behaviors may already have changed in our population, with individuals fearing to transmit deleterious mutations to their descendants. Most probands, however, consulted the oncogeneticist because they had cancer and few because of a family history of cancer. At their initial consultation, probands had generally completed their reproductive period (age at consultation 50 ± 13 years). Genetic consultation and mutation testing during the reproductive period was not available to older generations, and thus mutation status could not have conciously influenced their reproductive choices. With the advent of widespread BRCA testing for women and families at risk, current and future generations may however incorporate mutation status into their family planning.

Women affiliated to BRCA mutated families underwent 1/3 fewer miscarriages, in contrast to the observation of a matched case-control study including 3,485 BRCA carriers or non-carriers [25], where no difference in the rate of spontaneous abortions was found. Reporting of miscarriages is surely incomplete in our study, and no distinction was made between spontaneous abortion and stillbirth. In Europe, spontaneous abortion affects about 15 to 20% of all pregnancies [26–27]. In a great majority of cases, this event happens in the first weeks of pregnancy and may often not be perceived by parents. Reporting thus was likely to be heavily weighted toward stillbirths, defined as the death of the fetus during the third trimester of pregnancy [28]. The standard rate of stillbirths is evaluated at around 3% of all pregnancies [27]. We should thus expect about 2,090 stillbirths in our population instead of the 714 reported. Nevertheless, as this reporting takes place during pedigree building, before BRCA status is known, all groups were treated similarly and under-reporting should not be biased toward one or another group. Finally, the observed difference in family miscarriage rate could not be confirmed in directly tested individuals, because of their insufficient numbers.

The standard clinical parameters predicting BRCA mutations were validated in our pedigrees, showing our database and results are consistent with published data. The very large size of the database gave power and accuracy to the study. More than 12% of the regional population is included in the database, with pedigree data collected over decades in the same manner by the same geneticists. The limit imposed for this study of only analysing families with >5 members further contributed to the homogeneity of the study group, as most immigrant families were excluded by this critereon.

To conclude, BRCA mutations that survive selection pressure seem to provide significant fertility advantages. Fertility parameters should thus be considered as a novel source of data for future population research, in particular to shed new light on possible biological mechanisms of reproductive physiology. Also, it could help characterize new subgroups among families at cancer risk but where no BRCA mutation has been diagnosed, in particular distinguishing families where reproduction starts early and those where fertility advantage comes from a lower rate of miscarriage or a higher average number of offspring. These criterea may be useful for stratifying data produced in large-scale genomic analyses of low-penetrance genes.

## Supporting Information

S1 Data (ZIP)
